# Investigation of the Composition, Antimicrobial, Antioxidant, and Cytotoxicity Properties of *Salvia abrotanoides* Essential Oil

**DOI:** 10.1155/2022/9304977

**Published:** 2022-10-14

**Authors:** Zeinab Jaderi, Farideh Tabatabaei Yazdi, Seyed Ali Mortazavi, Arash Koocheki

**Affiliations:** Department of Food Science and Technology, Faculty of Agriculture, Ferdowsi University of Mashhad (FUM), Mashhad, Iran

## Abstract

Medicinal plants present promising attributes in traditional medicine based on earlier published documents. Most of the essential oils derived from vascular plants display a significant role in dealing with microbial and inflammation infections. This research aimed to provide informative knowledge about the composition, antimicrobial, and anticytotoxicity of *Salvia abrotanoides* essential oil. In this study, the chemical composition of *S. abrotanoides* was determined using FTIR and GC-MS analysis which demonstrated the significant number of monoterpenes in the constitutes. The antimicrobial activity of EO demonstrated a dose-related effect on several pathogenic bacteria and fungi; among bacteria, Gram-positive bacteria exhibited more sensitivity to the essential oil antimicrobial compounds. On the other hand, *S.* abrotanoides essential oil did not present antifungal activity as high as Fluconazole on Aspergillus niger and *Candida* albicans. The total phenolic and flavonoid content of essential oil were determined as 14.70 ± 1.4 mg·GA/g essential oil and 2.93 ± 0.41 mg Q/g essential oil, respectively. The antioxidant activity of essential oils was investigated, and it was not as high as positive controls. Moreover, the microscopic changes of *S. aureus* and *E. coli* were investigated using SEM images. The cytotoxicity potential of essential oil was evaluated on L929 and A459 cell lines and also it was estimated to be stronger on A459 cell line than that of L929.

## 1. Introduction

Essential oils (EO) represent the plant's secondary metabolites and are composed of several chemical structures [1]. These natural products mostly are considered potential bioactive resources with antimicrobial, antidiabetic, anticancer, and antioxidant activities, which result in exhibiting promising attributes in several scientific research in food, nutraceuticals, pharmaceuticals, cosmetics, and also emerging packaging industries due to their odor, flavor, fragrance, and biological capacities [2–5]. Essential oils are structurally composed of relatively small molecular weight compounds and usually have an aromatic odor [6]. Essential oils are divided into aliphatic, aromatics, and terpenoids and mainly are characterized by their low boiling point and high volatility [7, 8]. They generally are made of a different combination of mono- and sesquiterpenoids, benzenoids, phenylpropanoids, etc., with various biological effects on humans [9].

There has been an increasing interest in products that enjoy the advantages of antioxidant activities during the last few decades. Chemically synthesized antioxidants like butylated hydroxyanisole and butylated hydroxytoluene are provided in laboratories. Although these chemically synthesized antioxidants can promote human well-being, they are sometimes considered toxic to humans [10, 11]. Antibiotics present promising attributes in the development of human lives through attacking microorganisms. Nevertheless, antibiotic resistance has decreased the antibacterial efficiency of antibiotics. Many studies have been performed on the widely used natural sources of antibiotics, including medicinal herbs and plants, which display potential antimicrobial and antioxidant activities arising from their bioactive substances [12, 13].

Salvia represents the largest genus in the Lamiaceae family, with more than 980 species worldwide. *Salvia abrotanoides* (Kar.) Sytsma, formerly identified as *Perovskia abrotanoides*, is grown chiefly in Iran, Pakistan, and India [14, 15]. During the last decade, many studies have emphasized different attributes of the Salvia species due to its diverse biological activities. Indeed, the medicinal capacities of herbs and plants are mainly derived from their capacity to generate numerous bioactive secondary metabolites. Most of these metabolites have been reported for their advantageous antimicrobial, anti-inflammatory, antioxidant, antitumor, and insecticidal effects [16]. The advantageous bioactivities of Salvia are ascribed to the numerous chemical structures, like flavonoids and phenolic compounds, including salvianolic, caffeic, and rosmarinic acids, which usually have advantageous radical scavenging activities. In addition, these metabolites are composed of triterpenoids, monoterpenoids, diterpenoids, and sesquiterpenoids, which present different bioactivities [16, 17]. These miscellaneous chemical structures are considered for pharmaceutical and medicinal applications due to their antiviral, antioxidant, antibacterial, antiproliferative, antidiabetic, and anti-inflammatory characteristics [17–20]. Former phytochemical studies on *Salvia abrotanoides* were identified as irregular triterpenes, sesquiterpenoids, tetracyclic diterpenes, abietane, and icetexane diterpenoids [21–23]. The metabolites of S. abrotanoides have been used in traditional medicine, including the improvement of fever [24], antidiabetic [25], rheumatic pain [26], chronic hepatitis B virus (HBV), and leishmaniasis [27], and also other disorders like atherosclerosis, cardiovascular illness, liver, and cardiac fibrosis [28]. Likewise, it had particular role in Iranian traditional medical activity to heal leishmaniasis and skin disorders [21].

In this context, the essential oil of Salvia abrotanoides (Kar.) Sytsma has been provided, and then its antimicrobial activity against different pathogens has been assessed. Moreover, its chemical components and structures were studied by FTIR and gas chromatography. The antioxidant and anticytotoxicity were investigated, as well. To the best of our knowledge, this is the first time in the literature that cytotoxicity of this EO has been screened on A459 and L929 cell lines.

## 2. Materials and Methods

### 2.1. Extraction of Essential Oil

The plant was provided from a market in Khorasan, Iran and was identified by the taxonomist; deposited at the herbarium of the college of agriculture at the department of the Ferdowsi University of Mashhad. The plant's aerial parts, including leaves and flowers, have been dried and powdered by employing an electrical blender. Purification of essential oil was performed using a Clevenger-type apparatus for almost 3 hours. The purified oil was dried with anhydrous sodium sulphate and kept at 4°C for the following use [1].

### 2.2. Determination of Chemical Composition of S. abrotanoides EO

#### 2.2.1. Gas-Chromatography Assay

The purified essential oil was run in n-hexane for gas chromatography assay. The chemical composition analysis of the *S. abrotanoides* essential oils was performed on Agilent technologies gas chromatographs (GC) (KONIK HRGC 5000) paired with a 5975C mass spectrometer (MS) and quadripolar spectrophotometric detectors, enjoying an HP-5 MS capillary column (30 m × 0.25 mm, film thickness 0.25 *μ*m), and also equipped with a computer (Wiley 7n-library).

The temperature plan was as follows: 50°C (5 min), 50–250°C (3°C/min for 10 min), and 250°C (10 min), and also the temperature of the injection room was 250°C. Helium was applied in the role of the exporter gas at a 1.1 mL/min flow rate. 0.1 *μ*l of EO sample was injected manually, in split mode 1 : 50. Additionally, the MS operating parameters were set as follows: an ionization energy voltage of 70 eV, the scan range of 65/465 amu, and an ionization current of 150 *μ*A. Detection of EO composition was performed based on the retention time of n-alkanes (C8–C20) in the CP-Sil 8CB column, and was compared to the Adams 2007 references [29].

#### 2.2.2. Fourier Transform Infrared Spectroscopy (FTIR) Analysis

Initially, samples were kept at 25°C for 30 min before the FTIR analysis (an FTIR spectrometer (AVATAR 370 FTIR-made in the USA)). The FTIR spectrum of the extract was recorded at 500–4000 cm^−1^, the number of scans was 64, the resolution ratio was 4 cm^−1^, and the time to obtain scans was approximately 2 min [30]. In this assay, Folin-Ciocalteau (Sigma-Aldrich, Germany) reagent and gallic acid were applied as standards. Different concentrations (5–100 *µ*g/mL) of gallic acid and EO were blended with 2.5 mL (1N) Folin-Ciocalteau, and then shaken constantly for 2 minutes. Then, by adding 2 mL of sodium carbonate (Na_2_CO_3_), the mixture was incubated for 3 hours at room temperature. Both standard and samples' absorbance were measured at 760 nm using a spectrophotometer UV-VIS.

### 2.3. Total Flavonoid and Phenolic Content

Regarding assessing the Total Flavonoid Content (TFC), the method was centered on the development of a flavonoid-aluminum complex, and also quercetin was applied as a standard to make the calibration curve. This method involves diluting around 0.5 mL of EO with methanol, and then 5% sodium nitrite NaNO_2_ (0.15 mL) and aluminum chloride (AlCl_3_) are added. The mixture was placed in a stable for 10 min at room temperature. Next, 0.1 mL (1 M) NaOH was added and mixed well. Finally, the absorbance of the final mixture was determined at 510 nm by a spectrometer, UV-VIS. The Total Phenolic Content (TPC) of *S. abrotanoides* EO was assessed based on the method as reported by Borah et al. 2019 [31].

### 2.4. Evaluation of Antioxidant Activity

#### 2.4.1. DPPH Radical Scavenging Activity

The antioxidant capacity of the yielded EO of the *S. abrotanoides* was measured spectrophotometrically by 2.2-diphenyl-1-picrylhydrazyl (DPPH), which this method is established on the measurement of radical scavenging activity of EO. Ascorbic acid is the positive control in this part. The DPPH solution in ethanol (0.2 mmol/L) was provided. The EO was dissolved in ethyl acetate in varying concentrations; 3 mL of DPPH solution was added to the 4 mL of EO, at different concentrations, from 5 to 100 *µ*g/mL, and kept in the dark at room temperature for 30 min. Finally, the absorbance of the samples was measured at 517 nm using a UV-VIS spectrophotometer. The following equation, in which A blank is the absorbance of the control, was used to calculate the percentage of inhibition:

(1) $$\begin{array}{c} \left(I\%\right):I=\left(\frac{\left(A\;blank\right)-\left(A\;sample\right)}{A\;blank}\right)\times 100\;A:\text{absorbance}. \end{array}$$

The results were reported as an IC_50_ value expressing the dose required to make a 50% inhibition. A lower IC50 expresses a greater antioxidant capacity.

#### 2.4.2. *β*-Carotene/Linoleic Acid Bleaching (BCB) Assay

The antioxidant characterization of the tested oil was detected by this method. An assay was used, which was formerly described by Harkat-Madouri, et al., 2015, and is centered on the inhibition of the product of Linoleic acid oxidation, which is mainly considered a volatile compound. Briefly, the emulsion of *β*-Carotene-linoleic acid was prepared by mixing 200 mg of Twin 40, 1 mg cholochrome, 0.5 g *β*-Carotene, and 25 *μ*L of linoleic acid. Then, the emulsion was evaporated under low pressure at 40°C. Afterwards, 100 ml of distilled water was added, and 350 *μ*L of samples were mixed with 2.5 mL of the former solution, shaken, and incubated for 48 h at room temperature. BHT was used as the control in this assay. Finally, the absorbance was read at 490 nm [32].

The inhibition % was measured using the following equation:

(2) $$\begin{array}{c} \text{Antioxidant activity}\left(\%\right):\frac{A}{A_{0}}\times 100, \end{array}$$

A, absorbance of the sample after 48 h; A_0_ : Absorbance before the incubation.

### 2.5. Antimicrobial Activity

The antimicrobial activity of S. abrotanoides EO was monitored against four bacterial strains, including *S. aureus* (ATTCC 25923), *E. coli* (ATCC 25922), P.*aeruginosa* (ATTCC 278503), and S.*typhi* (ATCC 1609), and two fungal species including C. albicans (PTCC 5027) and A. Niger (ATCC 11414). The antibacterial potential was studied by the inhibition zone circle of disk diffusion agar assay in Mueller–Hinton agar (Sigma-Aldrich) plates and incubated for 24 h at 37°C. The potato dextrose agar was used to determine antifungal activity after 48 h at 28°C incubation. Kanamycin was administered as a positive control for antibacterial, and Fluconazole was used for antifungal potential [1]. Regarding WDA, the microbial suspension was dispersed onto petri dishes containing MHA medium by an L-shaped spreader. Subsequently, the EO (60 *μ*L) was loaded into the wells (6 mm diameter) on the medium surface. The dishes were placed at 37°C for 24 h. Finally, the inhibition zones around the wells were determined. Additionally, the MIC (Minimum Inhibitory Concentration), MBC (Minimum Bactericidal Concentration), and MFC (Minimum Fungicidal Concentration) were determined via dilution series assay centered on the method reported by Shakeri et al., 2014 [33].

### 2.6. Morphological Observation

In this assay, the mechanism of action of EO derived from *S. abrotanoides* was screened using SEM image analysis. Having said that, *S. aureus* (ATTCC 25923) and *E. coli* (ATCC 25922) were cultured at their MIC values in the shaking incubator at 37°C; the suspension was centrifuged for 5 min at 5000 g. Next, the adhered cells were removed via washing with ultrapure water and dried at room temperature. The washed *S. aureus* and *E. coli* were suspended in PBS (0.1 M, pH = 7) and filtered using a polycarbonate filter. Then, it was fixed in a 2.5% glutaraldehyde solution and saved at 4°C for 2 h. Afterwards, the solution was washed twice with ultrapure water and dehydrated in three steps with 30%, 70%, and 100% methanol. Finally, 20 *μ*L of the solution was spread on the aluminum surface, covered with gold particles, and investigated using a Scanning Electron Microscope (SEM) [34].

### 2.7. Cell Culture

Murine dermal fibroblasts (L929) and human lung cancer cell line (A459) were provided from the National Cell Bank of Iran, Pasteur Institute, Tehran, Iran, and kept in the T-25 flasks containing RPMI medium, supplemented with 1% glutamine, 10% FBS 1% (w/v), penicillin (100 U/mL), and streptomycin (100 g/mL). Then, cultures were incubated at 37°C under 5% CO_2_ in a humidified atmosphere [34].

### 2.8. Evaluation of Cytotoxic Activity

Cytotoxicity potential of the EO was determined using MTT (3-(4,5-dimethylthiazol- 2-yl)-2,5-diphenyl-2H-tetrazolium bromide) which is performed by reducing tetrazolium dye through a mitochondrial enzyme of viable cells. Briefly, L929 and A459 were seeded at a density of 10^5^ cells per well and 10^4^ cells per well in 96-well plates in the conditions described in the last section. The EO was suspended in culture media with 1% Twin 20 (v/v) and diluted to 0.9375–120 *μ*g/mL concentrations. The control cells were cultivated just with media, FBS, and Tween 20. The plates were incubated for 72 h, and cell proliferation by the MTT assay was assessed. Finally, the absorbance of the suspension was read at 570 nm via an ELISA reader (Convergent Technologies, Marburg, Germany) [33].

The results were reported as an IC50 value, expressing the dose required to achieve a 50% inhibition. The lower the IC50 express the greater the cytotoxic potential.

### 2.9. Statistical Analysis

The outcomes of this study were calculated as mean values and standard deviation (SD). One-way analysis of variance (ANOVA) and the Tukey test with *α* = 0.05 was completed by Minitab software.

## 3. Results and Discussion

### 3.1. Chemical Composition of *S. abrotanoides* EO by FTIR and GC-MS

**Figure 1:**
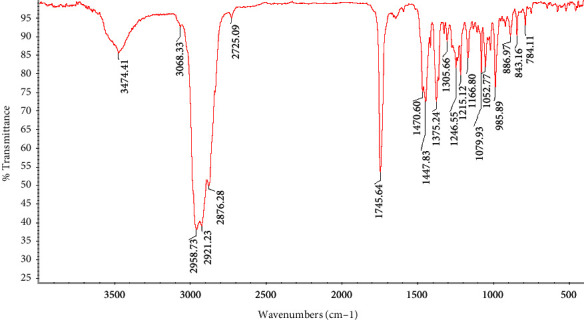
FTIR spectrum of *Salvia abrotanoides* essential oil.

The yield of the *Salvia abrotanoides* essential oil was determined as 1.7%. The functional groups of *S. abrotanoides* EO were conducted based on the vibrational frequencies in wavenumbers of the IR zone of the EO.  Figure 1 represents the FTIR spectra of the *S. abrotanoides* in spectral ranges of 4000 cm^−1^ to 400 cm−1. The broad absorption peak at 3474.41 cm^−1^ was consigned to the O–H stretching band and intermolecular bonds. There is a weak peak at 3068.33 cm^−1^, and the peaks between 3068.33 cm^−1^ and 2725.09 cm−1 represent the C–H stretching bond of alkanes and alkenes. A sharp peak at 1745.64 cm^−1^ corresponds to the vibration stretching of the aldehyde carbonyl C=O group. The peaks located at 1470.60 cm−1 and 1447.83 cm^−1^ are attributed to the C–H bending, a sharp peak at 1375.24 cm^−1^ characterized by O–H bending, and a peak at 1305.66 cm^−1^ show the alkanes CH2 bending. Peaks at 1246.55 cm^−1^ and 1215.12 cm^−1^ correspond to the aromatic acid ester C–O–C bond and C–OH of phenolic compounds. The peaks located in 1052.75 cm^−1^ and 1166.80 cm^−1^ are ascribed to the stretching C–O and C–C vibration, respectively. Finally, the presence of peaks at 985.89 cm^−1^ (C–H vibration band), two sharp peaks at 886.97 cm^−1^ and 843.16 cm^−1^ (C=C bending), and 784.11 cm^−1^ was occurred due to benzene ring = CH [30, 35, 36].

**Figure 2:**
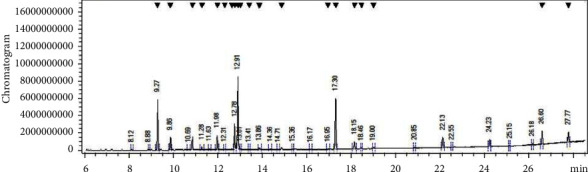
GC-MS spectrum of *Salvia abrotanoides* essential oil.

Essential oils are generally characterized by a high content of oxygenated monoterpenes, sesquiterpenes, diterpenes, and hydrocarbon monoterpenes [2]. A total of 17 compounds were identified from the GC-MS analysis of aerial parts of the S. abrotanoides EO, accounting for 99% of the whole EO, presented in Table 1, and Figure 2. The EO is principally composed of eucalyptol, camphor, and *α*-pinene (CID: 6654) with 23.5%, 20.2%, and 16.7%, respectively. Eucalyptol (C_10_H_18_O) is identified as a colorless liquid with a camphor-like odor, which is a flavoring agent and FDA-certified substance added to foods. It is chemically a naturally cyclic ether and a monoterpenoid. The peaks between 2900 and 3000 cm−1 in the FTIR spectrum could be ascribed to the presence of the eucalyptol (1,8-Cineole) compound. Camphor (C_10_H_16_O) (CID:2537) is the second abundant compound with cyclic monoterpene ketone (aromatic terpene ketone) structural attributes and is a natural monoterpenoid. The peak that occurred at 1745 cm^−1^ might be ascribed to the presence of the camphor component. D-Limonene, a monoterpene hydrocarbon (colorless mobile liquid with a pleasant lemon-like odor), is an oral dietary supplement containing a natural cyclic monoterpene (cyclohexane monoterpenes) with a formula of C_10_H_16_. Spectral peaks in 850–3000 might be related to this compound in EO components. 3-carene is another natural bicyclic monoterpene with a formula of C_10_H_16_ that could be a reason for peaks in 2800–2950 cm^−1^. Caryophyllene (C_15_H_24_) is a polycyclic sesquiterpene that could have peaks in 2850–3000 cm^−1^. It is also known as *β*-Caryophyllene and has a role as a nonsteroidal anti-inflammatory component, a fragrance, and an insect attractant. *β*-pinene is a natural plant metabolite, a bicyclic monoterpene and a monoterpene hydrocarbon. It is an isomer of pinene composed of an exocyclic double bond which might lead to the occurrence of peaks at 2850–3000 cm^−1^. Humulene (CID: 5281520), with a formula of C_15_H_24_, is an isomer of *α*- Humulene, is a monocyclic sesquiterpene and could have a vibrational peak in 2850–3000 cm^−1^. Camphene is a monoterpene hydrocarbon with a bicyclic skeleton (bicyclo [2.2.1] heptane) that could appear in peaks at 2900–3000 cm^−1^. Isoborneol (CID: 64685) (C_10_H_18_O) is a bornane monoterpenoid, bicyclic monoterpenes, and oxygenated monoterpenes. *β*-myrcene is an acyclic monoterpene with a formula of C_10_H_16_ that could have peaks in 850–950 cm^−1^. Cyclohexene (C_6_H_10_) is constituted of cycloalkene (cyclohexane) with a single double bond with probable peaks at 2750–2950 cm^−1^. *γ* -Terpinene is one of the three isomeric monoterpenes differing in the positions of their two double bonds, a minor compound that might have peaks at 2700–3050 cm^−1^. *β*-ocimene could have peaks at 2850–300 cm^−1^ is a minor component and considered monoterpenes. Meanwhile, the results indicated that *S. abrotanoides* could be considered a monoterpene hydrocarbon, oxygenated monoterpenes, and sesquiterpenoids-rich essential oil [37–39].

Ghavam et al., 2020 reported that GC analysis of EO from flowers and leaves of S. hydrangea showed 27 and 39 components, respectively. Also, they identified oxygenated sesquiterpenes and oxygenated monoterpenes as the most and lowest compounds in their study. Ghannadi et al. acknowledged 13 compounds using GC analysis of leaves of this plant species. Although some compounds are the same, the differences are likely because of chemotype, genetic, climate, and environmental conditions [40].

### 3.2. Total Phenolic and Flavonoid Content

Table 2 revealed the TPC and TFC values of S. abrotanoides EO. The TPC of EO was 14.70 ± 1.4 mg GA/g EO and the TFC was reported as 2.93 ± 0.41 mg Q/g EO. Quercetin and gallic acid were determined as TFC and TPC analysis standards, respectively. It was reported that the higher amount of phenolic content led to higher antioxidant and antimicrobial potential in EOs.

### 3.3. The Antioxidant Activity of S. abrotanoides

The antioxidant potential of *S. abrotanoides* EO was assessed using the two methods. The BHT and ascorbic acid were applied as controls in DPPH *β*-carotene/linoleic acid bleaching (BCB) assays. The related data are presented in Table 2. Although the DPPH assay indicated the IC50 values of 24.05 ± 0.91 *μ*g/mL as an antioxidant potential for EO, which means it has a high potential to reduce the value of free radicals in DPPH, it is lower (almost half of the positive control) than that of ascorbic acid as a control, and the values are different significantly (*p* < 0.5). On the other hand, the reducing power of the EO was 77.40 ± 1.27 *μ*g/mL, which is not as high as that of BHT. Kabouche et al., 2007 indicated that the acetone extract of S. barrelieri enjoys antioxidant and *β*-carotene/linoleic acid bleaching (BCB) activity stronger than that of positive controls like BHT and Vitamin E [41]. Ashraf et al., 2014 investigated the stem and leaves essential oils of Perovskia abrotanoides and reported that although leaves EO revealed a higher inhibition percent of 76.4% compared to the 66.1% of stem EO, the oils signified significantly lower antioxidant activity in comparison to those of BHT (90.4%) [42].

It is well documented that the antioxidant capacity of natural products is correlated to their phenolic and flavonoid content. Hazrati et al., 2020 reported that the antioxidant activity of EOs is ascribed to the oxygenated monoterpenes. In addition, based on several phytochemistry research, phenol and flavonoid content have many biological activities like antioxidant potential due to their hydroxyl groups [2].

### 3.4. In Vitro Cytotoxic Activity of EO

**Figure 3:**
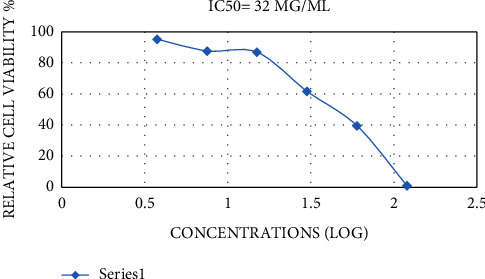
IC50 values of inhibition effects of *Salvia abrotanoides* essential oil on L929 cell line.

**Figure 4:**
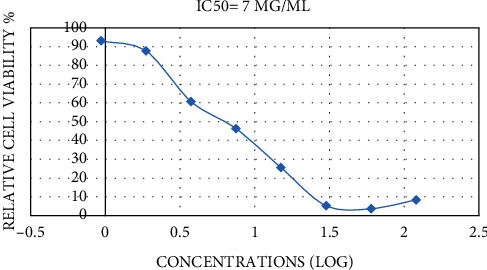
IC50 values of inhibition effects of *Salvia abrotanoides* essential oil on A459 cell line.

The MTT assay is a good indicator of cellular metabolic activity, cytotoxicity, proliferation, and cell viability. This is a colorimetric assay centered on the formation of purple formazan crystals by reducing yellow tetrazolium salt by the mitochondrial activity of cells [43]. This assay assessed the cytotoxic activity of *S. abrotanoides* EO against cultured murine dermal fibroblasts (L929) and human lung cancer cell line (A459). To the best of our knowledge, it is the first time that the impact of S. abrotanoides EO has been evaluated on L929 and A459 cell lines. The cell lines were exposed to an improving concentration of EO (0.9375–120 *μ*g/mL) for 72 h. There is a significant (*p* < 0.05) increase in the inhibition effect of EO on both cell lines as a function of improving the EO concentration, which is evident in IC50 values, which are presented in Figures 3 and 4. In light of the results, the inhibitory effect of EO saw a significant increase (*p* < 0.05) via increasing the EO concentration. However, there is a slight decrease in the inhibitory potential of EO on the A459 cell line in maximum concentration. The IC50 values were 32 and 7 *μ*g/mL for the L929 and A459 cell lines, respectively. The presented data revealed that *S. abrotanoides* EO have a more significant effect on the A459 cell line, and the L929 cell line is more resistant to the anti-inflammatory capacity of EO. The high monoterpenoid composition of S. abrotanoides EO makes it a cytotoxic appropriate compound. The anti-inflammatory capacity could be ascribed to the complex composition of oxygenated sesquiterpene and monoterpenes and sesquiterpenoids and monoterpenoids. Zhao et al., 2013 reported that the essential oil obtained from *A. anomala* revealed higher inhibition efficacy (8 times) on the MCF7 (human breast cancer cells) cell line in comparison to the A459 cell line [44]. Additionally, do Vale et al., 2019 evaluated the effect of *V. gardneriana* EO on L929 and HaCat and stated that 0.3–1.25% concentration indicated cytotoxic activity, but the morphological observation of cells showed that the cell structure did not change. This resulted that these cell lines are less susceptible to the *V. gardneriana* EO [34].

### 3.5. Antimicrobial Activity

Antimicrobial potential of *S. abrotanoides* essential oil was determined by the WDA and DDA method on food-borne pathogenic bacteria, including *S. aureus* (ATTCC 25923), *E. coli* (ATCC 25922), *P. aeruginosa* (ATTCC 278503), and *S. typhi* (ATCC 1609), and two fungal species including *C. albicans* (PTCC 5027) and *A. niger* (ATCC 11414). The results are presented in Tables 3 and 4 which indicated that the inhibition zone was increased significantly (*p* < 0.05) via improving the EO concentration from 25 to 150 mg/mL. Meanwhile, the lowest EO concentration (25 mg/mL) showed an inhibition effect just on *S. aureus*, which means its high susceptibility to the antimicrobial components of the EO. In contrast, the minimum EO concentration did not have an inhibition effect on other bacterial strains. In addition, the greatest EO concentration on *S. aureus* revealed the most extensive inhibition zone among other strains. These changes are likely to be correlated to the structural differences and composition of the lipopolysaccharide membrane [45]. Other studies have also reported the high resistance of Gram-negative bacteria to the natural antibacterial components [40]. The antifungal activity of EO also increased significantly (*p* < 0.05) with increasing the EO concentration in both of the studied fungi, and the *C. albicans* showed more susceptibility to the EO compounds, but the differences did not find significance.

In the DDA method, the inhibition zone on microbial strains was compared to the controls (antibiotics), kanamycin for bacteria and fluconazole for fungi. According to the results presented in Table 4, the inhibition zone was increased significantly (*p* < 0.05) with increasing the concentration. However, *S. aureus* indicated more inhibition rate than others, and *E. coli* showed a higher inhibition zone in comparison to other Gram-negatives. Moreover, antibiotics' antibacterial impact and maximum EO concentration saw a considerable change (*p* < 0.05). Indeed, there is a significant increase in kanamycin's antibacterial impact on *S. aureus* and *E. coli*. Nevertheless, the maximum concentration of EO presented the same antibacterial impact as the kanamycin on *P. aeruginosa* and S. Typhi, and no significant changes were depicted. Regarding the antifungal activity of EO compared to fluconazole, there is a significant increase in fluconazole antifungal potential, which means that S. abrotanoides EO did not present antifungal activity as high as fluconazole on *A. niger* and C. albicans.

The MIC, MBC, and MFC were assessed in 2–512 mg/mL (Table 5). Among the bacterial strains, *S. aureus* shows the lowest MIC and MBC and *E. coli* and *P. aeruginosa* indicated the maximum MIC (64). Also, the maximum MBC was depicted for *P. aeruginosa* and *S. typhi*. The MIC for *A. niger* and C. albicans were the same, but the MFC for C. albicans was greater (16 mg/mL compared to 8 mg/mL), which means that C. albicans is more persistent to the antifungal compounds of EO. C. albicans is one of the potent pathogenic fungi and is identified as a severe danger to human health. The results revealed that EO presents a more inhibitory effect against C. albicans and *A. niger*, demonstrating promising attributes to be identified as a natural antifungal agent. The antimicrobial activity of EO arises from its volatile compounds detected by GC-MS. The major group of monoterpenes has a diverse structural characterization and has a vital role in the antimicrobial activities of essential oils. Ghavam et al., 2020 demonstrated that S. hydrangea EO presented antibacterial activity against Gram-positive bacteria, including *S. aureus*, S. epidermidis, and B. subtilis. Nonetheless, it was lower than that of gentamycin and rifampin. While Sonboli et al., 2009 reported a significant antibacterial activity of S. hydrangea EO against S. epidermidis and B. subtilis, Kotan et al., 2008 presented a weak antibacterial activity against *S. aureus* [40]. Moreover, it was claimed that antimicrobial activity of the essential oils usually arises from *α*-pinene and *β*-pinene, which are abundant with a portion of 16.7% and 4.7% in this study.

**Figure 5:**
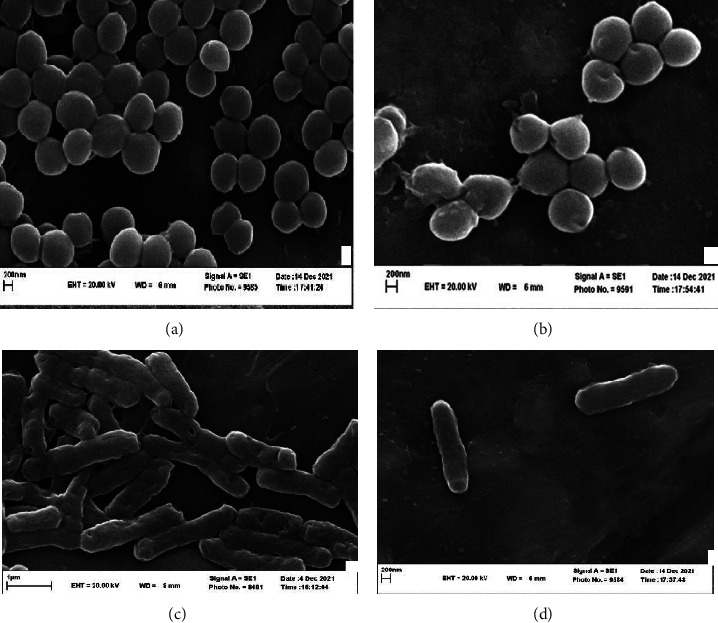
SEM images of *S. aureus* and *E. coli* treatment at MIC concentration with *Salvia abrotanoides* essential oil. (a) *S. aureus* without treatment. (b) treated S *aureus*. (c) *E. coli* without treatment. (d) Treated E *coli*.

The SEM images of the treated *S. aureus* and *E. coli* in their MIC against the untreated strains are provided in Figure 5. It is well observed that the presence of the EO antibacterial compounds leads to the destruction of the cell wall of the strains, which can result in cell lethality (antibacterial effect).

## 4. Conclusion

Essential oils are plant secondary metabolites and are recognized as vital representatives of plant defense procedures against pathogens which can be varied via their habitat, the generated organs, and climate conditions. In this study, the chemical composition of the EO derived from S. abrotanoides was investigated using FTIR and GC-MS analysis. Afterwards, the total phenolic and flavonoid content of EO and its antioxidant activity were identified. The antimicrobial capacity of EO on pathogenic microorganisms was evaluated, and it was found that the antifungal activity of EO is higher than its antibacterial potential using MBC and MFC assays. The results indicated a higher inhibitory effect of EO on the A459 cell line compared to L929. In light of the results of this study, it could be claimed that this essential oil could be a potent representative of phenolic compounds, which would make it a valid alternative to some antimicrobial and anti-inflammatory drugs and result in coping with antimicrobial resistance.

## Figures and Tables

**Table 1:** Chemical composition of *S. abrotanoides* Gas chromatography mass spectrometry. Retention indices (RIs) relative to n-alkanes (C6–C40) on the same methyl silicone capillary column.

No.	Compound	Retention times (RT)	Area (%)
1	*α*- pinene	9.27	16.7
2	Camphene	9.86	4.2
3	*β*-pinene	10.86	4.7
4	*β*-myrcene	11.29	1.2
5	3-Carene	11.98	5.1
6	Cyclohexene	12.31	0.2
7	o-cymene	12.63	0.3
8	D- limonene	12.78	8.5
9	Eucalyptol	12.91	23.5
10	*β*- ocimene	13.01	0.4
11	*γ* -terpinene	13.86	0.7
12	Camphor	17.30	20.2
13	Isoborneol	18.15	3.2
14	Terpinen-4-0l	18.46	0.3
15	*α*-Terpineol	19.01	0.5
16	Caryophyllene	26.60	4.9
17	Humulene	27.77	4.4
	Total		99

**Table 2:** Total phenolic and flavonoid content, and antioxidant activity of *S. abrotanoides.*

sample	DPPH IC50 (*μ*g/ml)	*β* Caroten/Linoleic acid (%inhibition)	TPC (Total phenolic content) mg·GA/g essential oil)	Total flavonoids (mg Q/g) essential oil
BTH	24.05 ± 0.91	^b^77.40 ± 1.27^b^	14.70 ± 1.4	2.93 ± 0.41
Ascorbic acid	—	92.15 ± 1.77^a^	—	—
Values that are followed by different letters within each column are significantly different (*p* < 0.05).	12.25 ± 0.49^a^	—	—	—

Zone of inhibition (mm).

**Table 3:** Antimicrobial activity of *S. abrotanoides* using WDA method in different concentrations.

Microorganisms concentrations (mg/mL)	Well diffusion *agar*			
	25 mg/ml	50 mg/ml	100 mg/ml	150 mg/ml
*Bacteria*				
*S.aureus* ATTCC 25923	8.10 ± 0.14^c^	8.95 ± 0.49^bc^	10.45 ± 0.63^ab^	11.90 ± 0.56^a^
*E.coli* ATCC 25922	**—**	7.00 ± 0.28^b^	7.85 ± 0.63^ab^	9.65 ± 0.49^a^
*P.aeruginosa* ATTCC 27853	—	6.60 ± 0.14^b^	7.45 ± 0.49^ab^	8.65 ± 0.49^a^
*S.typhi* ATCC 1609	—	6.75 ± 0.21^b^	7.35 ± 0.21^ab^	8.30 ± 0.42^a^

*fungi*				
*A.Niger* ATCC 11414	9.60 ± 0.56^b^	10.50 ± 0.28^ab^	11.15 ± 0.21^a^	11.85 ± 0.21^a^
*C. albicans* PTCC 5027	10.15 ± 0.21^b^	10.65 ± 0.21^b^	11.35 ± 0.21^ab^	12.15 ± 0.49^a^

Note. The different letters (a–d) in a row indicate the significant differences at *p* < 0.05, based on one-way ANOVA analysis. Zone of inhibition (mm).

**Table 4:** Antimicrobial activity of *S. abrotanoides* using DDA method in different concentrations.

Microorganisms Concentrations (mg/mL)	Disk diffusion *agar*				
	25 mg/ml	50 mg/ml	100 mg/ml	150 mg/ml	Antibiotics
*bacteria*					Kanamycin
*S.aureus* ATTCC 25923	7.50 ± 0.28^d^	8.65 ± 0.354^cd^	9.90 ± 0.42^bc^	11.40 ± 0.56^b^	22.80 ± 0.28^a^
*E. coli* ATCC 25922	—	6.65 ± 0.35^c^	7.85 ± 0.21^c^	9.15 ± 0.35^b^	17.00 ± 0.28^a^
*P.aeruginosa* ATTCC 27853	—	6.40 ± 0.14^b^	7.15 ± 0.21^ab^	8.15 ± 0.35^a^	7.55 ± 0.21^a^
*S.typhi* ATCC 1609	—	6.50 ± 0.14^b^	7.10 ± 0.28^b^	8.30 ± 0.84^ab^	9.85 ± 49^a^

*fungi*					*Fluconazole*
A.Niger ATCC 11414	8.95 ± 0.70^d^	9.35 ± 0.21^cd^	9.95 ± 0.21^c^	11.05 ± 0.35^b^	25.55 ± 0.21^a^
C. albicans PTCC	50279.40 ± 0.14^c^	9.90 ± 0.14^c^	10.85 ± 0.49^bc^	11.90 ± 0.56^b^	24.05 ± 0.35^a^

Note. The different letters (a–d) in a row indicate the significant differences at P<0.05, based on one-way ANOVA analysis.

**Table 5:** Antimicrobial activity of MIC, MBC, and MFC *of Salvia abrotanoides* essential oil on some pathogenic bacteria.

Microorganisms	MIC (mg/ml)	MBC/MFC (mg/ml)
*S.aureus* ATTCC 25923	8	16
*E. coli* ATCC 25922	64	64
*P.aeruginosa* ATTCC 27853	64	128
*S.typhi* ATCC 1609	32	128
*C. albicans* PTCC 50274	8	
*A.Niger* ATCC 11414	4	16

MIC and MBC/MFC of *Salvia abrotanoides* essential oil on some pathogenic strain causing infection.

## Data Availability

All of the data are presented in the manuscript in tables and figures.

## References

[1] Liu T., Lin P., Bao T. (2018). Essential oil composition and antimicrobial activity of Artemisia dracunculus L. var. qinghaiensis YR Ling (Asteraceae) from Qinghai-Tibet Plateau. *Industrial Crops and Products*.

[2] Hazrati S., Govahi M., Sedaghat M., Beyraghdar Kashkooli A. (2020). A comparative study of essential oil profile, antibacterial and antioxidant activities of two cultivated Ziziphora species (Z. clinopodioides and Z. tenuior). *Industrial Crops and Products*.

[3] Kacjan Marsic N., Necemer M., Veberic R., Poklar Ulrih N., Skrt M. (2019). Effect of cultivar and fertilization on garlic yield and allicin content in bulbs at harvest and during storage. *Turkish Journal of Agriculture and Forestry*.

[4] Coşge Şenkal B., Uskutoglu T., Cesur C., Ozavci V., Dogan H. (2019). Determination of essential oil components, mineral matter, and heavy metal content of Salvia virgata Jacq. grown in culture conditions. *Turkish Journal of Agriculture and Forestry*.

[5] Pirnia M., Shirani K., Tabatabaee Yazdi F., Moratazavi S. A., Mohebbi M. (2022). Characterization of antioxidant active biopolymer bilayer film based on gelatin-frankincense incorporated with Ascorbic acid and Hyssopus officinalis essential oil. *Food Chemistry X*.

[6] Zhang J., Huang R. Z., Cao H. J. (2018). Chemical composition, in vitro anti-tumor activities and related mechanisms of the essential oil from the roots of Potentilla discolor. *Industrial Crops and Products*.

[7] Bajer T., Surmova S., Eisner A., Ventura K., Bajerova P. (2018). Use of simultaneous distillation-extraction, supercritical fluid extraction and solid-phase microextraction for characterisation of the volatile profile of Dipteryx odorata (Aubl.) Willd. *Industrial Crops and Products*.

[8] Tian H., Zhao H., Zhou H., Zhang Y. (2019). Chemical composition and antimicrobial activity of the essential oil from the aerial part of Dictamnus dasycarpus Turcz. *Industrial Crops and Products*.

[9] Baser K. H. C., Buchbauer G. (2009). *Handbook of Essential Oils: Science, Technology, and Applications*.

[10] He F., Wang W., Wu M. (2020). Antioxidant and antibacterial activities of essential oil from Atractylodes lancea rhizomes. *Industrial Crops and Products*.

[11] Mishra P. K., Shukla R., Singh P., Prakash B., Dubey N. K. (2012). Antifungal and antiaflatoxigenic efficacy of Caesulia axillaris Roxb. essential oil against fungi deteriorating some herbal raw materials, and its antioxidant activity. *Industrial Crops and Products*.

[12] Ceylan S., Cetin S., Camadan Y., Saral O., Ozsen O., Tutus A. (2019). Antibacterial and antioxidant activities of traditional medicinal plants from the Erzurum region of Turkey. *Irish Journal of Medical Science*.

[13] Bączek K. B., Kosakowska O., Przybyl J. L. (2017). Antibacterial and antioxidant activity of essential oils and extracts from costmary (Tanacetum balsamita L.) and tansy (Tanacetum vulgare L.). *Industrial Crops and Products*.

[14] Hu G.-X., Takano A., Drew B. T. (2018). Phylogeny and staminal evolution of Salvia (lamiaceae, nepetoideae) in east asia. *Annals of Botany*.

[15] Drew B. T., Gonzalez-Gallegos J. G., Xiang C. L. (2017). Salvia united: the greatest good for the greatest number. *Taxon*.

[16] Jassbi A. R., Zare S., Firuzi O., Xiao J. (2016). Bioactive phytochemicals from shoots and roots of Salvia species. *Phytochemistry Reviews*.

[17] Zimowska B., Bielecka M., Abramczyk B., Nicoletti R. (2020). Bioactive products from endophytic fungi of sages (Salvia spp.). *Agriculture*.

[18] Jang S.-I., Jeong S. I., Kim K. J. (2003). Tanshinone IIA from Salvia miltiorrhiza inhibits inducible nitric oxide synthase expression and production of TNF-alpha, IL-1beta and IL-6 in activated RAW 264.7 cells. *Planta Medica*.

[19] Wang X., Wei Y., Yuan S. (2005). Potential anticancer activity of tanshinone IIA against human breast cancer. *International Journal of Cancer*.

[20] Kim E. J., Jung S. N., Son K. H. (2007). Antidiabetes and antiobesity effect of cryptotanshinone via activation of AMP-activated protein kinase. *Molecular Pharmacology*.

[21] Alizadeh Z., Donadio G., Farimani M. M., Parisi V., Ebrahimi S. N., De Tommasi N. (2021). Two seco-norabietane diterpenoids with unprecedented skeletons from the roots of Salvia abrotanoides (Kar.) Sytsma. *Phytochemistry*.

[22] Alizadeh Z., Farimani M. M., Parisi V., Marzocco S., Ebrahimi S. N., De Tommasi N. (2021). Nor-abietane diterpenoids from perovskia abrotanoides roots with anti-inflammatory potential. *Journal of Natural Products*.

[23] Tabefam M., Farimani M., Danton O. (2018). Antiprotozoal diterpenes from Perovskia abrotanoides. *Planta Medica*.

[24] Sadeghi Z., Masullo M., Cerulli A., Nazzaro F., Farimani M. M., Piacente S. (2020). Terpenoid constituents of Perovskia artemisioides aerial parts with inhibitory effects on bacterial biofilm growth. *Journal of Natural Products*.

[25] Matkowski A., Miroliaei M., Aminjafari A., Slusarczyk S., Nawrot-Hadzik I., Rahimmalek M. (2017). Inhibition of glycation-induced cytotoxicity, protein glycation, and activity of proteolytic enzymes by extract from Perovskia atriplicifolia roots. *Pharmacognosy Magazine*.

[26] Jiang H.-L., Wang X. Z., Xiao J. (2013). New abietane diterpenoids from the roots of Salvia przewalskii. *Tetrahedron*.

[27] Jiang Z. Y. (2015). Abietane diterpenoids from perovskia atriplicifolia and their anti-HBV activities. *Bulletin of the Korean Chemical Society*.

[28] Hashemifar Z., Rahimmalek M. (2018). Genetic structure and variation in Perovskia abrotanoides Karel and P. atriplicifolia as revealed by molecular and morphological markers. *Scientia Horticulturae*.

[29] Adams R. P. (2007). *Identification of Essential Oil Components by Gas Chromatography/Mass Spectrometry*.

[30] Falah F., Shirani K., Vasiee A., Tabatabaee Yazdi F., Alizadeh Behbahani B. (2021). In vitro screening of phytochemicals, antioxidant, antimicrobial, and cytotoxic activity of Echinops setifer extract. *Biocatalysis and Agricultural Biotechnology*.

[31] Borah A., Paw M., Gogoi R. (2019). Chemical composition, antioxidant, anti-inflammatory, anti-microbial and in-vitro cytotoxic efficacy of essential oil of Curcuma caesia Roxb. leaves: an endangered medicinal plant of North East India. *Industrial Crops and Products*.

[32] Harkat-Madouri L., Asma B., Madani K. (2015). Chemical composition, antibacterial and antioxidant activities of essential oil of Eucalyptus globulus from Algeria. *Industrial Crops and Products*.

[33] Shakeri A., Khakdan F., Soheili V., Sahebkar A., Rassam G., Asili J. (2014). Chemical composition, antibacterial activity, and cytotoxicity of essential oil from Nepeta ucrainica L. spp. kopetdaghensis. *Industrial Crops and Products*.

[34] Vale J. P. C. d., Ribeiro L. H., Vasconcelos M. A. (2019). Chemical composition, antioxidant, antimicrobial and antibiofilm activities of Vitex gardneriana schauer leaves’s essential oil. *Microbial Pathogenesis*.

[35] Alizadeh Behbahani B. (2020). Chemical composition and antioxidant, antimicrobial, and antiproliferative activities of Cinnamomum zeylanicum bark essential oil. *Evidence-based Complementary and Alternative Medicine*.

[36] Coates J. (2000). *Interpretation of Infrared Spectra, a Practical Approach*.

[37] *PubChem, P.C. Compound Summary. ncbi*.

[38] Samantha E., Gad P. J. H. (2005). *Encyclopedia of Toxicology*.

[39] Sharifi-Rad J., Sureda A., Tenore G. (2017). Biological activities of essential oils: from plant chemoecology to traditional healing systems. *Molecules*.

[40] Ghavam M., Manca M. L., Manconi M., Bacchetta G. (2020). Chemical composition and antimicrobial activity of essential oils obtained from leaves and flowers of Salvia hydrangea DC. ex Benth. *Scientific Reports*.

[41] Kabouche A., Kabouche Z., Ozturk M., Kolak U., Topcu G. (2007). Antioxidant abietane diterpenoids from Salvia barrelieri. *Food Chemistry*.

[42] Ashraf S. N., Zubair M., Rizwan K. (2014). Compositional studies and Biological activities of Perovskia abrotanoides Kar. oils. *Biological Research*.

[43] Marinas I. C., Oprea E., Buleandra M. (2021). Chemical composition, antipathogenic and cytotoxic activity of the essential oil extracted from amorpha fruticosa fruits. *Molecules*.

[44] Zhao J., Zheng X., Newman R. A., Zhong Y., Liu Z., Nan P. (2013). Chemical composition and bioactivity of the essential oil of Artemisia anomala from China. *Journal of Essential Oil Research*.

[45] Shirani K., Shahidi F., Mortazavi S. A. (2020). Investigation of decontamination effect of argon cold plasma on physicochemical and sensory properties of almond slices. *International Journal of Food Microbiology*.

